# Metabolome searcher: a high throughput tool for metabolite identification and metabolic pathway mapping directly from mass spectrometry and using genome restriction

**DOI:** 10.1186/s12859-015-0462-y

**Published:** 2015-02-25

**Authors:** A Ranjitha Dhanasekaran, Jon L Pearson, Balasubramanian Ganesan, Bart C Weimer

**Affiliations:** Center for Integrated BioSystems, Computer Science Department, Utah State University, Logan, 84322-8700 USA; Western Dairy Center, Department of Nutrition, Dietetics, and Food Sciences, Utah State University, Logan, 84322-8700 USA; University of California, Davis, School of Veterinary Medicine, 1089 Veterinary Medicine Dr., VM3B, Room 4023, Davis, CA 95616 USA; Linda Crnic Institute for Down Syndrome, Department of Pediatrics, School of Medicine, University of Colorado Denver, 12700 E 19th Avenue, Aurora, CO 80045 USA; Spillman Technologies, 4625 West Lake Park Blvd, Salt Lake City, UT 84120 USA

## Abstract

**Background:**

Mass spectrometric analysis of microbial metabolism provides a long list of possible compounds. Restricting the identification of the possible compounds to those produced by the specific organism would benefit the identification process. Currently, identification of mass spectrometry (MS) data is commonly done using empirically derived compound databases. Unfortunately, most databases contain relatively few compounds, leaving long lists of unidentified molecules. Incorporating genome-encoded metabolism enables MS output identification that may not be included in databases. Using an organism’s genome as a database restricts metabolite identification to only those compounds that the organism can produce.

**Results:**

To address the challenge of metabolomic analysis from MS data, a web-based application to directly search genome-constructed metabolic databases was developed. The user query returns a genome-restricted list of possible compound identifications along with the putative metabolic pathways based on the name, formula, SMILES structure, and the compound mass as defined by the user. Multiple queries can be done simultaneously by submitting a text file created by the user or obtained from the MS analysis software. The user can also provide parameters specific to the experiment’s MS analysis conditions, such as mass deviation, adducts, and detection mode during the query so as to provide additional levels of evidence to produce the tentative identification. The query results are provided as an HTML page and downloadable text file of possible compounds that are restricted to a specific genome. Hyperlinks provided in the HTML file connect the user to the curated metabolic databases housed in ProCyc, a Pathway Tools platform, as well as the KEGG Pathway database for visualization and metabolic pathway analysis.

**Conclusions:**

Metabolome Searcher, a web-based tool, facilitates putative compound identification of MS output based on genome-restricted metabolic capability. This enables researchers to rapidly extend the possible identifications of large data sets for metabolites that are not in compound databases. Putative compound names with their associated metabolic pathways from metabolomics data sets are returned to the user for additional biological interpretation and visualization. This novel approach enables compound identification by restricting the possible masses to those encoded in the genome.

## Background

Bacterial metabolism impacts almost every aspect of our life. Microbial metabolism was exploited by early human civilization to create fermented foods and beverages [1,2]. The oldest known metabolically derived products from microbes include bread, cured meats, cheese, and beer [2-4]. Currently, metabolic engineering for the production of pharmaceuticals and bioactive compounds is giving way to discovery of novel metabolic pathways for production of alternative fuels [5-7]. Burgeoning needs to produce novel antibiotics for disease treatment and health supplements, such as amino-sugars and vitamins, also represent the metabolic end products that are genome encoded of an organism [8-11].

The virulence of bacterial pathogens is closely linked to their metabolism during infection, which is leading to metabolomic disease biomarkers that is pushing the boundaries of robust methods to quickly identify high throughput metabolomic data [12,13]. Cumulatively, the unusual metabolic networks of organisms in ecological niches are renewing interests in metabolites that highlight the lack of high throughput analysis tools for rapid compound identification when the compound is not included in a database. Unfortunately, rapid identification of multiple metabolites simultaneously is also lacking. However, if one considers an organism’s genome to be a database of possible metabolic pathways and metabolite production, it enables customization of MS output analysis based on a specific organism. Approaching the genome as a metabolite database is being done using metabolic reconstruction methods in KEGG and Pathway Tools.

The metabolism of an organism changes during growth, survival, and persistence via complex gene expression changes. In many cases, metabolism begins with the transport of chemically diverse molecules for integration into biologically functional blocks. An organism’s metabolic capability can be envisaged as a highly interconnected network of enzymatic reactions that provide energy, intermediates for macromolecular biosynthesis, cellular signaling, regulation of stress, and control of oxidation/reduction to ensure growth or survival [14]. Highly tuned regulatory mechanisms to modulate the metabolic network via gene expression and enzyme attenuation are needed to quickly adapt to local environmental changes. Evolution of genetic control and gene acquisition are critical to ensure the organism’s survival in the near- and long-term [15]. Adaptation and genetic evolution results in new metabolic nodes in the interconnected network that modifies the intermediate and end product metabolism [14,16]. Of recent interests, metabolic engineering is largely dependent on understanding the metabolic network to regulate production of specific low molecular weight end products that often accumulate.

Low molecular weight metabolites, usually <1,000 Da (small molecules; Table 1), including sugars, lipids, fatty acids, amino acids, nucleotides, vitamins, and co-factors are usually the targets of metabolomics, which have bioactivity and lead to biomarker profiles (www.metacyc.org; [17]). An organism’s metabolic demands are met by catabolism of complex macromolecules to the constituent small molecules (e.g. polysaccharides to sugars) or digestion of the molecules themselves (e.g. vitamins and amino acids) to end products. The products of catabolism are reassembled through anabolic pathways into macromolecules of the organism to derive energy, oxidation/reduction regulation, pH control, and to maintain membrane potential that fuels transport functions. During growth catabolic and anabolic processes are regulated both genetically and biochemically to maintain a balance between growth and survival [18,19]. All of these activities are encoded in the genome, which provides an inherent genetic database of the possible metabolic compounds that an organism can produce during changing growth conditions.

**Table 1:** **Metabolite distribution by molecular mass across metabolic encyclopaedias**

**Molecular weight range (Da)**	**Number of compounds in Metacyc**	**Number of compounds in KEGG**
50-99	254	267
100-199	1,731	2,402
200-299	1,050	2,614
300-399	842	2,575
400-499	461	1,150
500-599	311	719
600-699	169	337
700-799	199	201
800-899	198	232
900-999	133	160
>1,000	119	224
Total	5,467	10,881

Metabolomics aspires to identify all the metabolites produced by an organism [20,21]. However, large data sets, limited identification databases, and limited MS parameters to differentiate small molecules are stumbling blocks for metabolomic analysis, which in turn limits the subsequent bioinformatic analysis and construction of biologically informative models [16,21]. Currently, NMR is of limited use for high throughput small molecule identification due to the lack of sensitivity and limited throughput, but is useful to elucidate the structures of unique metabolites [22,23]. However, NMR is very useful to track the metabolic fate of a small molecule with isotope labels, which provides information for a handful of metabolites once the entire compound list is narrowed to a specific set of metabolic intermediates [18]. Other post-separation detection techniques like photometric, electrochemical, and fluorescent detection are actively used to identify specific metabolites at a substantially reduced analytical scale, but the need to identify the set of compounds produced is overwhelmingly changing the goals of metabolite analysis [24-26]. Conversely, MS analysis, in addition to metabolic tracking estimates the masses of hundreds to thousands of small molecules within minutes and provides information on their relative levels in the sample [27-29], making it very useful for high throughput metabolome analysis. However, it lacks specific information as to the identity of the small molecules, which highlights the need to have curated databases for compound identification [21].

One approach to overcome the need to identify important molecules uses principal component analysis (PCA) to find changes with a specific treatment. From MS data acquisition this produces a reduced list of small molecules that are tagged as biomarkers [30,31]. Often the diagnostic peak is an unknown compound that is difficult to identify. Subsequently, more complex chemical analysis is used to determine the elemental composition of these biomarkers, which requires additional time, expertise, and often multiple instrumentation capabilities [32,33]. Biomarker identities are subsequently validated by standard compound injection to produce a compound library [22]. While this statistics-driven analytical approach favors method development for MS it ignores the underlying biochemistry and the importance of relatively minor changes of small molecules that can sometimes lead to misinterpretation of the biological impact with new small molecule production. This is especially prevalent for key metabolite classes like hormones, vitamins, and enzyme co-factors where small changes regulate large scale proteomic and metabolic fluctuations [23]. One way to overcome this limitation is to use tools that include all possible putative compounds generated directly from matched compound identities prior to statistical analysis. Subsequently, a significant list of putative compounds can be used for metabolic mapping to facilitate biological identity by linking compound identities to metabolic pathways and routes. Feist et al. [24] review the reconstruction approach with specific attention to metabolite identification.

Unfortunately, metabolite identification from hundreds to thousands of masses by searching a large compound database is a slow process that is ill defined relative to the specific search criteria that provides confident compounds assignments. GC-MS analysis often identifies compounds by comparison of MS spectra with large, well-established compound libraries (www.nist.gov). Such compound libraries for LC-MS analysis are available for only a small set of masses and are tightly linked to the LC conditions. Large compound databases such as Pubchem (http://pubchem.ncbi.nlm.nih.gov) and Chemspider (http://www.chemspider.com) allow searches of single masses and other query types, but they do not allow queries from large lists of masses or connect putative compounds to metabolic pathways. However, as the query list expands, as it does in metabolome data sets, data analysis using single queries becomes unrealistic for a timely and accurate analysis.

Multiple software suites are available for compound identification of mass spectrometry-based metabolite data that use mass spectral deconvolution and matching to reference databases. Some examples of full-fledged independent platforms are MetSign [25], MZmine 2 [26], MAVEN [27], and XCMS^2^ [28], whereas MS Excel templates such as IDEOM [29], R packages like AStream [30] and MAIT [31], and web-applications like METLIN [32], XCMS Online [33], and MZedDB [34] are also available as web services. These tools offer either statistical or structural analyses of small molecule MS data and extract information from metabolic databases to create a list of compounds for their own localized database. For example, MetSign’s compound database is formed from the cumulative compound collection of KEGG, HMDB, and LIPIDMAPS databases, MZmine 2′s collection is from KEGG [35], HMP, and Pubchem compound, MAVEN uses KEGG, whereas MAIT, IDEOM and ASTREAM use unspecified databases. However, downstream of compound identification, they ignore the underlying biology and do not offer a mechanism to map the data back to the metabolic pathways. Further, they lack the flexibility of implementing user-defined parameters for database searches, as for example, electrospray ionization (ESI) parameters that are predefined in METLIN and MZedDB [34].

Querying large compound databases that contain millions of non-biological molecules can impede a researcher’s ability to overlay a metabolic context onto metabolomic data [36]. Biologists are producing data at rates that outstrip the ability of analysts to examine the data set to uncover the biological importance. To keep pace with metabolome analysis, high throughput bioinformatic tools that bring compound identity and pathway relevance together to the biologist are crucial. This can be accomplished with: a) automated searches of metabolic databases to retrieve putative compound identification, b) large scale queries be performed seamlessly with MS output, c) provide users the flexibility of using multiple query types, and d) map query results to metabolic pathways, hence allowing data to be analyzed in a biological context.

The availability of over 1,000 annotated microbial genome sequences enables bioinformatic reconstruction (biocyc.org) of an organism’s metabolic capability via the genome, which provides a broad network of metabolism that can be used to predict small molecule production [27,28]. Consequently, recent efforts have focused on uncovering the metabolic networks in many different biological systems [19,37]. Genome reconstructions of the metabolic pathways coupled to analytical methods, such as liquid chromatography (LC), gas chromatography (GC) and capillary electrophoresis with nuclear magnetic resonance spectroscopy (NMR) and mass spectrometry (MS) produces a new method to leverage genomic sequence to provide putative compound identification quickly [27,38].

In this study, a user-friendly web-based application called Metabolome Searcher to retrieve a list of small molecules identifications based on chemical formula, SMILES structure, and the monoisotopic mass was created using an organism’s genome as a putative compound database. While single queries can be directly entered multiple queries with one or more query types can also be done using a text file containing the query list. One or more reference databases can be selected from the list against which the queries are performed. The output connects small molecules in a sample to metabolic databases via embedded links to specific metabolic pathways. The Metabolome Searcher’s output allows researchers using metabolome data from different technologies to group the compound identifications into metabolic information so as to uncover the relevant biological function with multiple chemical criteria.

## Methods

### The ProCyc webserver

We currently house a metabolic database webserver called ProCyc (www.usu.edu/westcent/procyc), which is an implementation of the Pathway Tools webserver (SRI Bioinformatics, Menlo Park, CA) with our own manually and automatically curated metabolic databases of interest. ProCyc houses over 47 metabolic database reconstructions of different classes of bacteria including probiotics, lactic acid bacteria, pathogens, and environmental bacteria that were reconstructed locally. The MetaCyc database and Human metabolism database are part of the basic installation of Pathway Tools software. Some of the reconstructed databases and the tier I/II databases of the basic software were used to exemplify the Metabolome Searcher implementation. This particular platform was chosen for its flexibility to immediately incorporate user-discovered pathways into the right metabolic databases.

### Metabolic reference database creation

A Metabolic Reference Database (MRDB) of an organism is a flat file (tab-delimited, plain text file) that initially contains only the compound name, molecular formula, molecular weight, SMILES structure, and the respective pathways for all compounds extracted from Pathway Tools Pathway/Genome Database (PGDB) of that organism. The script to create the MRDB communicated with Pathway Tools [17] via the PerlCyc module (v1.1; www.arabidopsis.org/biocyc/perlcyc). The same approach was used to create an additional non-redundant database using Metacyc [17] and KEGG [35] (Table 2). The reference monoisotopic masses of individual elements were obtained from a publicly available compilation (Scientific Instrument Services, Inc., Ringoes, NJ; www.sisweb.com). Using the monoisotopic masses of individual elements, the monoisotopic masses of all compounds in the MRDB in their charged and neutral states were calculated based on their formulae. The MRDB was then modified to include the calculated monoisotopic masses, which is queried for compound identification and pathway mapping via the Metabolome Searcher’s web interface.

**Table 2:** **Organism-specific and general metabolic reference databases available for the Metabolome Searcher**

**Organism**	**ProCyc database**	**Metabolic reference database**
*Escherichia coli* K12	EcoCyc	*E. coli* K12
*Escherichia coli* O157:H7	Ecoo157Cyc	*E. coli* O157:H7
*Homosapiens*	HumanCyc	*Homo sapiens*
KEGG Compounds		KEGG Compounds
*Lactococcus lactis* ssp. *lactis* IL1403	LlactisCyc^1^	*L. lactis* ssp. *lactis* IL1403
*Lactococcus lactis* ssp. *cremoris* SK11	LaccremoCyc^1^	*L. lactis* ssp. *cremoris* SK11
*Lactobacillus acidophilus* NCFM	LbacidCyc^1^	*Lb. acidophilus* NCFM
*Lactobacillus johnsonii* NCC 533	LbjohnCyc^1^	*Lb. johnsonii* NCC 533
*Lactobacillus plantarum* WCFS1	LbplanCyc^1^	*Lb. plantarum* WCFS1
*Listeria monocytogenes* EGDe	LmonoCyc^1^	*Listeria monocytogenes*EGDe
*Mycobacterium bovis* AF2122/97	MbovisCyc^1^	*M. bovis* AF2122/97
MetaCyc	MetaCyc	MetaCyc
*Staphylococcus aureus* Mu50	SaureusCyc^1^	*S. aureus* ssp. *aureus* Mu50
*Saccharomyces cerevisiae* S288C	YeastCyc^2^	*S. cerevisiae* S288C
*Salmonella enterica* ssp. *enterica* serovar Typhimurium LT2	Styp99287Cyc^3^	*Salmonella typhimurium* LT2

^1^PGDBs reconstructed, curated and hosted in ProCyc.

^2^Obtained from the Yeast genome database.

^3^Downloaded from the Pathway Tools registry of PGDBs.

### Query input

**Figure 1:**
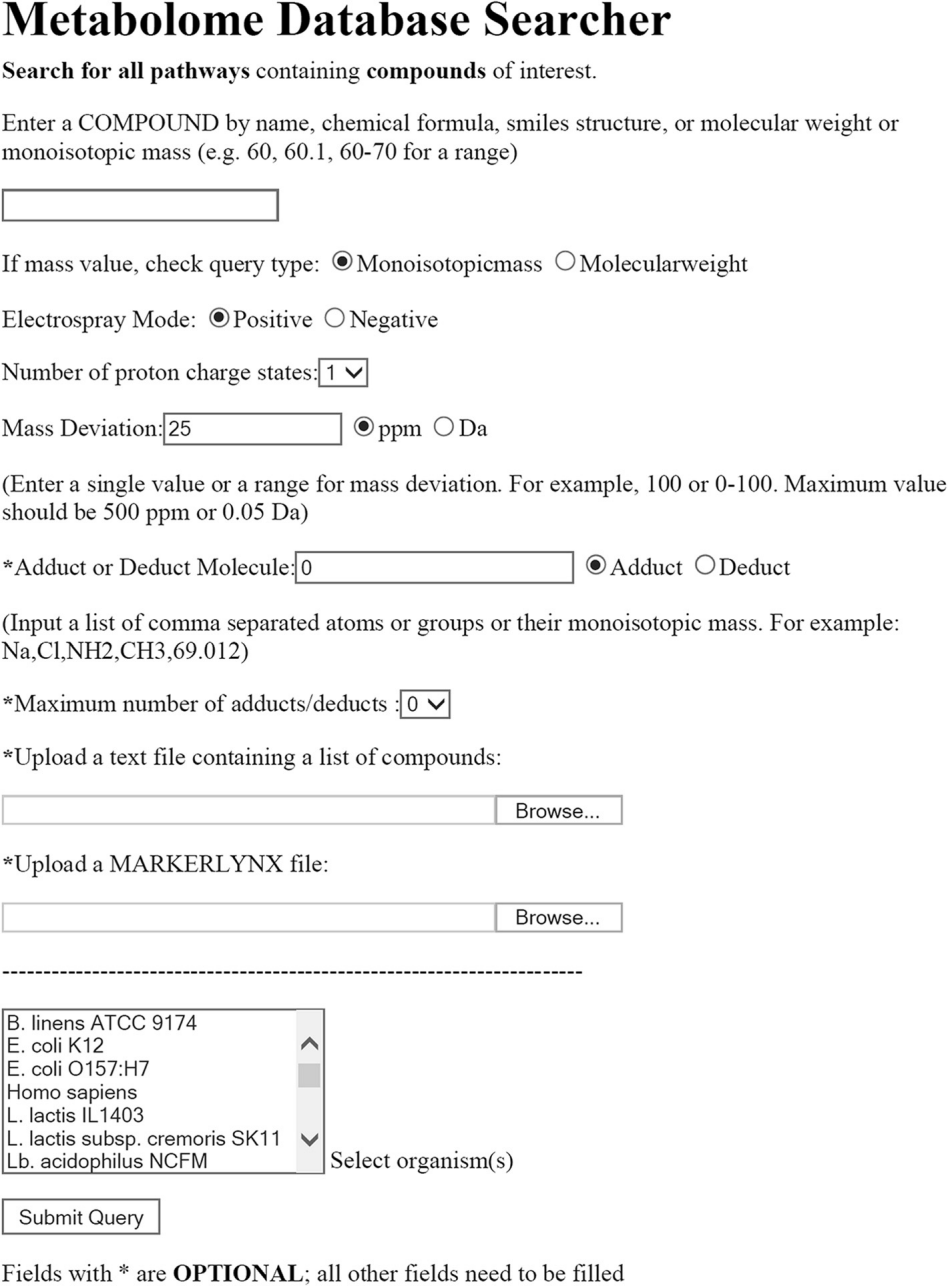
**Metabolome searcher user interface screenshot.**

The Metabolome Searcher allows the user to enter a single query by typing the name, formula, molecular/monoisotopic mass or SMILES structure, or multiple queries by uploading a query list within a file (Figure 1). This file contains masses and intensities of compounds as a tab-delimited text file. For mass searches, whether from a single entry or a file, the user selects the type (molecular weight or monoisotopic mass). Most MS systems contain software that enables data export to a text or an Excel file [25]. We used the QTof system (QTof Premier, Waters, MA) with MarkerLynx software for marker identification and analysis to test this approach. A MarkerLynx-derived text file was used without modification for the Metabolome Searcher query by submitting the file under the “MarkerLynx file” input on the interface (Figure 1). Alternately, analysis of output from other MS systems can be done using the “text file” option (Figure 1). While using the text file option, query values of any type, whether masses or specific compound names or a mixture of query types, were listed in the first column of the query file. Any headers, empty lines, and non-query values in the first column were removed prior to submission of data as a text file for matching. For both the file options, other information like statistics, marker quality, peak areas, peak heights, and concentrations across experiments and replicates were still retained in the file.

### Compound identification for MS analysis

For compound identification from monoisotopic masses, the user specifies the acceptable deviation from the theoretical masses (ppm or Da, under “Mass deviation”; Figure 1), the ionization mode (positive or negative, under “Electrospray mode”; Figure 1), the maximum number of charges (0-5; under the “Number of proton charge states”; Figure 1), and adducts (mass or formula; optional; under “Adduct or Deduct molecule” and “Maximum number of adducts/deducts”; Figure 1). The deviation value allows the software to obtain matches for queried masses within an acceptable range to narrow or expand the putative identification list. Acceptable mass deviation values may be experimentally determined or obtained from the literature based on a particular instrument and operating conditions [37].

Typically during MS analysis the molecules are detected by prior ionization with or by removal of protons (positive and negative mode, respectively) [23]. The MS settings are optimized to mainly produce singly charged ions. However, a molecule may still carry multiple charges depending on the MS settings [38]. The user can verify the charge state of compounds contained in the input list to recalibrate the MS settings by selecting different charge states during multiple search sessions.

Positively charged ionic species, such as sodium (Na+) and potassium (K+), or negative species, such as chloride (Cl−) and formate (HCOO−), are also used during ionization due to their abundance in a sample. The addition of ionic species or adducts during ionization shifts the observed monoisotopic mass from that of the intact molecule plus/minus a proton [38]. These adducts can be specified either as individual elements or as partial functional groups in the “Adduct or Deduct molecule” textbox (Figure 1). Similar to adducts, if the user wishes to specify fragments lost during ionization or fragmentation the “Deduct” option can be selected. The user can also provide more than one adduct or deduct in the textbox simultaneously and specify the number of maximum possible adducts or fragments (“Maximum number of adducts/deducts” option).

### Database selection

MRDBs that contain metabolites from different PGDBs or the KEGG database along with calculated monoisotopic masses are used for the queries. MRDBs are included for user selection from the ones listed on the interface (Table 2) wherein the user can select single or multiple MRDBs for searching (Figure 1). If the user intends to query known metabolic pathways in an organism, the organism-specific MRDBs are provided for more specific and narrow options of possible compounds due to the known annotated pathways. However, if the intent is to discover new pathways unknown in a particular system, but identified in other organisms, or if an organism without a pre-constructed MRDB is being studied, the user can select a genotypically related organism’s MRDB or the MetaCyc MRDB for matching. A user-generated PGDB can also be incorporated as an MRDB using the scripts defined above prior to the user defined query. The MRDBs were created in a flat file format to reduce complexity in processing and data handling such that newer MRDBs for other organisms can always be created in a consistent format and readily incorporated as per the user’s need. Pathway Tools was selected as the main metabolic database platform to create MRDBs and link back to PGDBs due to its interactive features and user-level flexibility for metabolic database development and curation of whole genome PGDBs [17], while queries of an MRDB for the KEGG database [39] are also supported.

### Database searching

**Figure 2:**
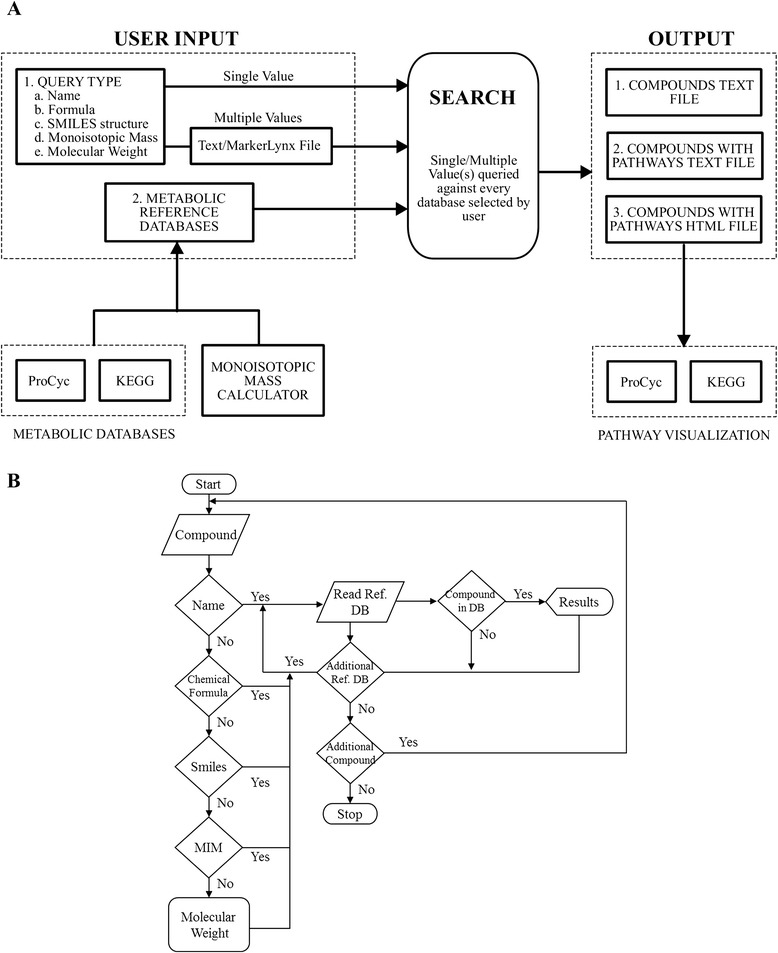
**Diagram of the work flow and search operations that underly metabolome searcher to return compounds and pathways. (A)** Metabolome searcher workflow. **(B)** flowchart of the search operations to find matching compounds.

Once a text query has been submitted, the Metabolome Searcher determines whether a text input is the name of a compound, its chemical formula or its SMILES structure independent of any specifications. After the query is classified into the specific type, information of the corresponding type in the MRDB is used for matching (i.e. names-to-names, formulae-to-formulae, and masses-to-masses) (Figure 2). All matches obtained within the parameters specified for searches are provided in the output files for viewing and analysis.

### Output generation

**Figure 3:**
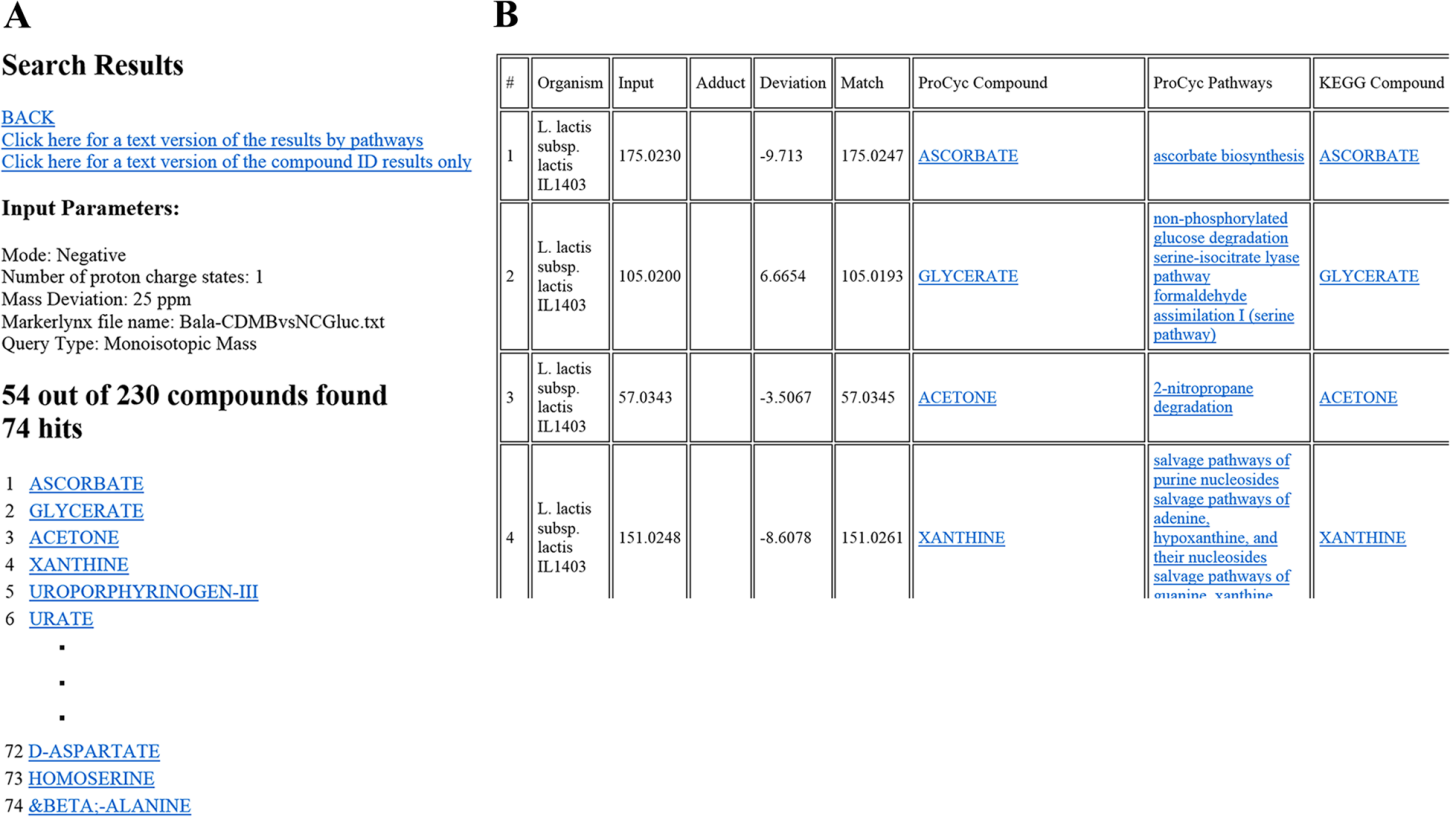
**Screenshot of metabolome searcher’s output. (A)** The top portion of the HTML results page and **(B)** the body of the HTML file demonstrate that sections containing queries, matches, compound and pathway links, and other data and information are provided with in the output.

After entering a single query or uploading a query file and specifying the MRDBs along with other MS analysis parameters, the user submits the query. The queries are matched against the MRDBs and the output files are created. Query parameters are printed at the top of all the output files to ensure that the parameters submitted by the user were used for searching the database (Figure 3A).

Three different output files are provided as the result of the analysis, one HTML and two text files. The two text files are embedded as links at the top of the HTML page (Figure 3A) that the user can download. One text file (“compounds file”) lists only the matched compounds without any metabolic pathway information, while the other (“pathways file”) repeats each compound’s data by all the pathways that it belongs to as a metabolite.

All scripts were written in Perl (v5.8.6; www.perl.org). The scripts and the metabolic reference databases for Metabolome Searcher are hosted in an Apple XGrid computational cluster (Panther OS 10.3.9) at the Western Dairy Center at Utah State University as well as University of California, Davis. Web pages for data input and output were created using Perl CGI.

### MS data validation

#### Chemical standards preparation

All compounds used were purchased from Sigma-Aldrich (St. Louis, MO). A chemically defined medium described previously by Ganesan et al. [18] was prepared as a complex mixture for testing Metabolome Searcher’s performance. The major components of this medium are 20 amino acids, sodium chloride, citrate, phosphate, 3-(N-morpholino) propane sulfonic acid (MOPS), vitamin solution (containing 15 different compounds), and glucose. Individual standard solutions of selected amino acids, glucose, citrate, and MOPS were also used for molecule identification.

### Mass spectrometry

Separation and analysis of standard compound mixtures were done at the mass spectrometry facility in the CIB. The samples were separated by liquid chromatography (2795 LC system; Waters) prior to introduction by electrospray into the mass spectrometer (QTof Premier; Waters) as described by Mortishire-Smith et al. [40]. Briefly, the separation was done for 10 min using a linear gradient of water:acetonitrile from 0-95% using a Symmetry C18 column (Waters). After introduction into the MS by electrospray, the molecules were detected using both positive and negative electrospray conditions, with calibrated settings recommended by the manufacturer. The QTof instrument was operated in W mode throughout MS analysis. For both positive and negative electrospray analysis, the conditions were: desolvation temperature of 250°C, source temperature of 120°C, cone voltage of 40 V, and collision energy of 4 eV. Data acquisition was performed for a mass range of 50–1,000 Da. After acquisition the data were centroided [40] using 1 ng/μl leucine-enkephalin infused at 10 μl/min as a reference, with an m/z of 556.2771 in positive mode and m/z of 554.25 in negative mode. In order to subtract background from the LC column and sample matrix, HPLC-grade water (Thermo Fisher Scientific Inc., Waltham, MA) was injected into the MS as a negative control. All samples were analyzed in technical duplicates.

Peak detection, intensity extraction, and normalization were performed using MarkerLynx software (Waters) to obtain monoisotopic masses and molecule retention times. In this study, only the monoisotopic masses of the markers were used for database searches. The Metabolome Searcher does not support any data analysis of the concentrations or relative measures of compound levels obtained from MarkerLynx.

## Results and discussion

Metabolomic assessment provides a list of compounds that facilitates the estimation of metabolic flux through both single pathways and networks [41,42]. Metabolome analysis enables determination of abiotic conditions and genetic regulation of metabolic networks. To achieve these purposes a tool that rapidly determines the compound identity, pathways, and metabolic networks was needed [43,44]. The tool accepts queries from common data types and facilitates data integration from independent sources into a unified compound identification and pathway-mapping scheme. To our knowledge such a tool is not available. The Metabolome Searcher addresses these purposes by receiving input from the user, querying the user-selected metabolic reference database(s), and displaying the generated output for further biological interpretation (Figure 3).

Of the Metabolome Searcher’s outputs, the compounds file is useful when the user plans to conduct compound classification, data clustering, principal component analysis, analysis of variance, or graphical visualization. The pathways file allows the users to sort the data by pathways and facilitates interpretation of metabolic flux and pathway connections to determine if a compound is an intermediate or an end product. The main feature of the HTML output is that it lists and links compounds to all metabolic pathways in which the metabolite is involved (Figure 3). These links help the user understand the role of that particular metabolite in the organism’s metabolic network. The user can click on any one of these links that will navigate them to the PGDBs curated and hosted at ProCyc. The user need not repeat queries on the Metabolome Searcher as the HTML file contains the links to the pathways associated with the returned putative compound IDs. To facilitate obtaining the standard chemicals for verification of retention times, CAS IDs of compounds (where available) are also included in a separate column in the output file.

### Verification

For names, formulae, and SMILES structures, any partial matches will also be detected and listed. For example, a query of the word string “glucose” against the MetaCyc database will identify D-glucose and an additional 52 hits (data not shown) that also include alpha-methyl-glucose, NDP-Glucoses, and all other molecules that contain the substring “glucose” in the name. String matching offers the user the ability to obtain partial matches and allows additional control over the query specificity and flexibility for unknown pathways. In most cases, if the specific MetaCyc compound names are used, the results will be restricted to one hit. Searching of word strings was implemented in order that even if other data sources such as GC-MS and LC-MS/MS were provided after identification using other software suites, or even data from standard GC or HPLC analyses based on extractions and retention times under certain conditions was provided, the data can be mapped to metabolites and pathways.

**Figure 4:**
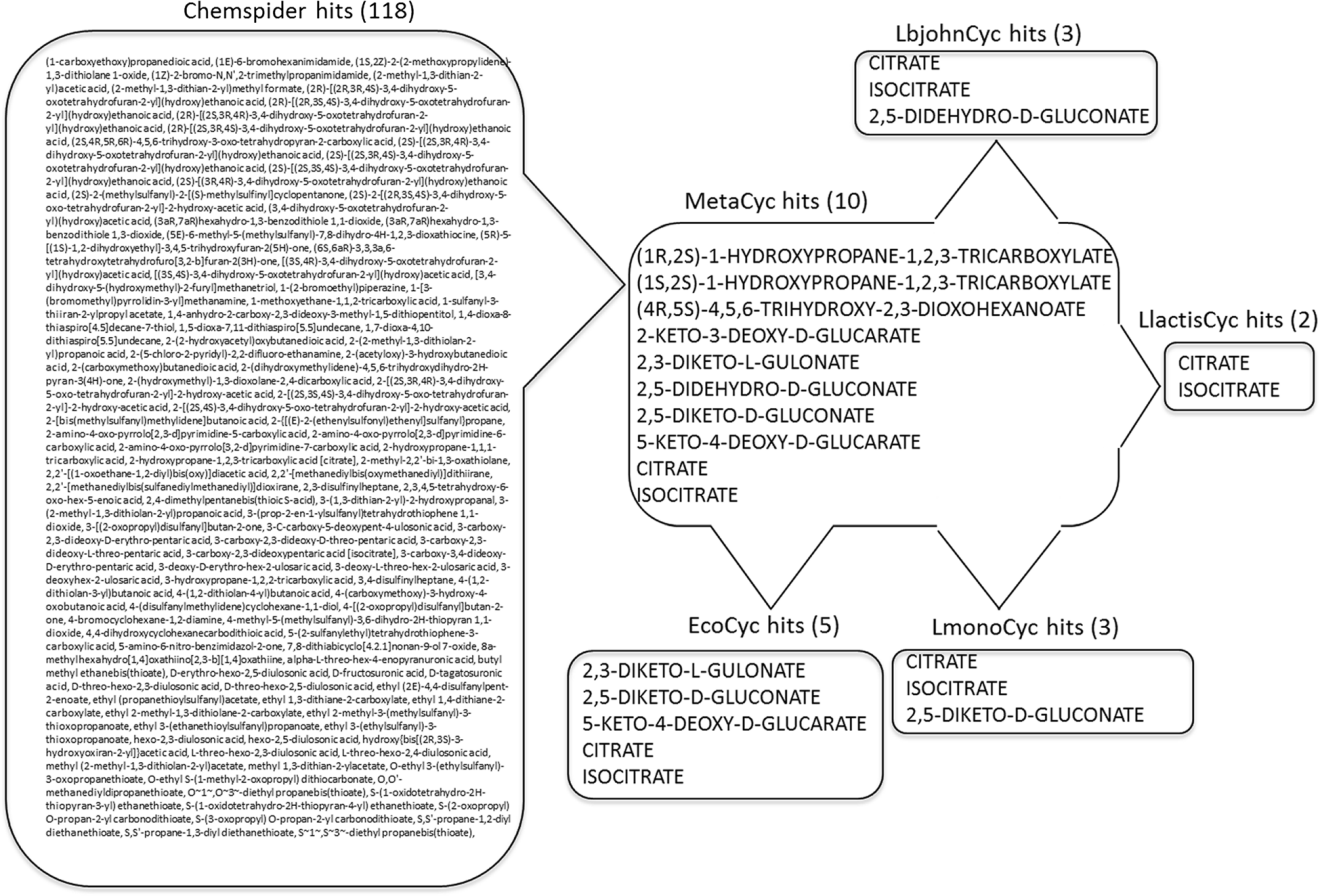
**Comparison of results from chemical vs metabolic databases with the monoisotopic mass of isocitrate (192.027 ± 0.001 Da) as the query.** Hits to an encyclopedia of genes (MetaCyc), *E. coli* (EcoCyc), *Listeria monocytogenes* (LmonoCyc), *Lactococcus lactis* IL1403 (LlactisCyc), and *Lactobacillus johnsonii* (LbjohnCyc) databases were used to demonstrate multiple genome-restrictions using Metabolome Searcher.

Compound identification from LC-MS or NMR spectrometry data has proven to be a challenge to biologists because the compound databases are limited, especially with respect to the compounds that a specific organism can produce. Based on the user selection of MRDB(s) in Metabolome Searcher, the number of hits is refined and is metabolically relevant to the organism under study, which provides a basis for biological conclusions to be drawn. As an example of the convenience provided by Metabolome Searcher by implementing genome restriction, we initially queried the MetaCyc MRDB with the monoisotopic mass of isocitrate as the search query and used the results for further narrowing the hits by querying organism-specific MRDBs. These genome-restricted results were compared to those hits obtained by querying the monoisotopic mass of isocitrate using Chemspider (Figure 4). The ChemSpider query returned 118 possible compound identifications that included non-biological compounds and required extensive analysis outside the query system to derive possible identifications whereas querying the MetaCyc MRDB provided hits that included 10 compounds with similar monoisotopic masses to that of isocitrate. Each genome (i.e. organism) further reduced the hits to 2–5 compounds that reflected the genetic differences in metabolism, all of which were related to citrate. Combining genome restriction with the MS compound list refined the possible identification list to a low number of compounds that was reasonable for empirical confirmation.

The interface and search function were verified by accessing the database search function using known exact masses and a data set generated from a known mixture of compounds (i.e. a chemically defined bacterial growth medium) from LC-MS output. The resulting markers exported into a MarkerLynx format text file was used to query the compound identification using Metabolome Searcher. All the main ingredients of the growth medium represented in the MetaCyc MRDB were detected during the search (Table 3). MOPS, a buffering salt, was used as a negative control for the chemical challenge, which was done by excluding it from the MetaCyc MRDB. Interestingly, after excluding MOPS, some of the query masses also matched multiple metabolites, many of which were isomeric forms of the metabolites being tested. This allowed further restriction of identification to narrower ranges of mass deviation to obtain better accuracy. However, in nearly 90% of compounds identified the number of hits was limited to <5 metabolites, thus aiding the directed development of protocols for further compound identification. This approach enabled detection of common starting substrates for metabolism and verified that if the compound was in the database, Metabolome Searcher found it.

**Table 3:** **Summary of hits to selected compounds from a chemically defined growth medium determined from a query of monoisotopic masses using the Metabolome Searcher**

**Compound**	**Detected by mass match**	Number of additional hits*	Number of non-isomeric additional hits*
Tyrosine	Yes	29	25
Pyridoxamine	Yes	0	0
Lysine	Yes	4	1
Thymidine	Yes	0	0
Pyridoxal	Yes	8	5
Glycine	Yes	1	0
Dihydrouracil	Yes	4	0
Arginine	Yes	2	1
Threonine	Yes	7	0
Alanine	Yes	5	0
Serine	Yes	1	0
Asparagine	Yes	5	0
Histidine	Yes	3	1
Tryptophan	Yes	2	1
Aspartate	Yes	2	0
Glutamate	Yes	6	0
Guanosine	Yes	0	0

*Additional hits include all individual compounds that match the query using the provided query settings; non-isomeric hits only include compounds that do not share the same empirical formula.

### Uses of metabolome searcher

**Figure 5:**
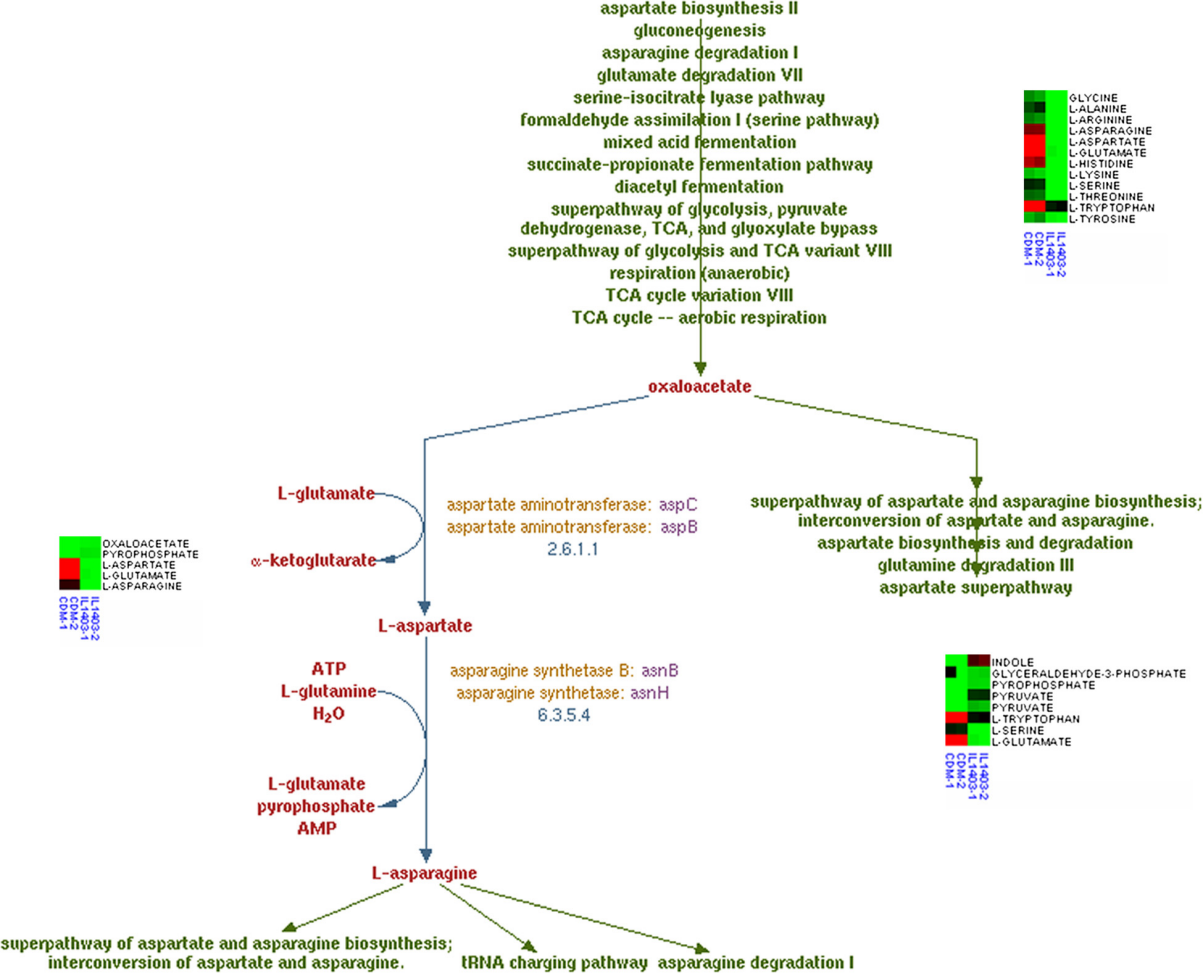
**Pathway assembly of metabolome searcher output with heat maps of LC/MS data from the compounds file and the pathways file (tryptophan biosynthesis and asparagine biosynthesis I).** This output is obtained by clicking on the pathway link for “asparagine biosynthesis I” inside the HTML file that brings up the pathway page at ProCyc. The heat maps are color-coded with green being 0, black the median value, and red the highest value within that data set. The heatmap number of 1 was at the initial time and number 2 was 24 hours later, which demonstrates the change in metabolites, from the MS list and visualized with the pathway and concentration.

An example demonstration of the Metabolome Searcher for microbial metabolomics was by collecting metabolomics profiles for both sterile chemically defined media and spent media collected after inoculation with the bacterium *Lactococcus lactis* IL1403 for 16 h. Metabolomics profiles were collected by LC-MS analysis in both positive and negative electrospray modes for the same samples and the masses obtained from MarkerLynx were queried against the *L. lactis* IL1403 MRDB (Table 2). After overlaying the compound identifications we quickly inferred changes in compound classes, such as amino acids (Figure 5), by sorting the compounds file, or pathways file that changed during growth of *L. lactis* IL1403 (Figure 5). This example demonstrated that Metabolome Searcher performed the intended search and enabled the biological meaning to rapidly assign the identified compounds using constructed databases from metabolic reconstruction maps.

## Conclusions

The Metabolome Searcher provides an automated tool to identify metabolites from MS analyses from metabolic reconstruction of specific genomes. This approach couples long lists of masses to specific genomic-based metabolites for identification and subsequent visualization via metabolic pathways. The tool is flexible so that query types can use many types of data that include names, molecular formulae, or SMILES structures, and monoisotopic masses that are entered singly or in bulk as a text file. The matches to queries are then presented as results along with other input parameters that the user included in the query and the pathways in which the matched metabolites are involved. The versatility of accepted query types and the provision of pathways mapped to queries are unique to the Metabolome Searcher. The Metabolome Searcher’s utility and flexibility facilitates rapid advances from metabolomics to biological comprehension.

### Availability of supporting data

Metabolome Searcher can be accessed at http://procyc.westcent.usu.edu/cgi-bin/MetaboSearcher.cgi.
